# Health-related quality of life in patients with primary immunodeficiency disease

**DOI:** 10.1186/s13223-015-0092-y

**Published:** 2015-09-29

**Authors:** Fonda Jiang, Troy R. Torgerson, Andrew G. Ayars

**Affiliations:** University of Washington, Seattle, WA USA; Seattle Children’s Hospital, Seattle, WA USA; Center for Allergy and Inflammation UW Medicine at South Lake Union, 850 Republican Street, Seattle, WA 98109-4725 USA

**Keywords:** Intravenous immunoglobulin, Immunoglobulin therapy, Immunoglobulin G, Immunoglobulin treatment, Primary immunodeficiency disease, Health-related quality of life

## Abstract

Primary immunodeficiency disease (PIDD) with hypogammaglobulinemia is characterized by recurrent and severe bacterial infections and IgG replacement is the standard of care in many of these patients. Health-related quality of life (HRQOL) is becoming increasingly recognized as a factor that affects patient well-being and treatment preferences. In an effort to better understand what factors affect HRQOL in patients with PIDD, we reviewed the published literature that used standardized instruments for the measurement of HRQOL. We investigated HRQOL in PIDD patients compared with normal controls and patients with other chronic diseases; we also investigated the impact of treatment administration on patient satisfaction. The most commonly encountered health-related quality of life instruments were the child heath questionnaire parental form 50, short form 36, PedsQL 4.0, Lansky’s play performance scale, and Life Quality Index. Patients with PIDD scored significantly lower on many of the instruments compared with normal controls. Also, while it appears that many patients appreciate home-based and subcutaneous IgG replacement therapy, patient satisfaction ultimately involves various clinical factors and individual patient preferences. By further analyzing what factors impact HRQOL, therapy adjustments can be made to maximize patient well-being and minimize disease impact on daily functioning.

## Background

Primary immunodeficiency disease (PIDD) is a group of genetic disorders characterized by increased susceptibility to infection. The incidence of PIDD has increased markedly in the last 40 years, likely secondary to an expanded understanding of the genetic basis and defective immunological mechanisms responsible for PIDD [1–3]. The exact prevalence of PIDD is not known. Estimates state 1 in 2000 children, 1 in 1200 for all persons, and 1 in 600 households (yielding a total population estimate of between 150,000 and 360,000 persons) in the US are diagnosed with PIDD [1]. One survey estimates the most frequently occurring form of PIDD—isolated IgA deficiency—occurs in approximately 1 in 163 individuals in Spain [4]. PIDD can affect patients at any age necessitating long-term therapy with IgG replacement therapy.

Survival is often considered the most important outcome in study design; however, the impact of illness on health-related quality of life (HRQOL) is receiving increasing recognition. HRQOL measurements are becoming important when analyzing not only the effect of the disease on patient well-being, but the satisfaction of patients with specific treatment regimens. Because many patients with PIDD require therapy indefinitely, the form of administration and setting where the product is administered are important factors that can significantly affect HRQOL. Specific measures to evaluate the outcome of an illness or its treatment include quantity and quality of life, and economic cost. The following is a review of the medical literature that measured HRQOL in PIDD patients.

### Health related quality of life (HRQOL) indicators

Outcomes of morbidity and mortality are considered the gold standard in most clinical studies; however, the impact of illness on HRQOL has received increasing recognition. One challenge is that HRQOL is difficult to conceptualize and even more difficult to measure. The World Health Organization defines quality of life as an individual’s perception of their position in life in the context of the culture and value systems in which they live and in relation to their goals, expectations, standards and concerns [5]. It is a broad ranging concept affected by the person’s physical health, psychological state, level of independence, social relationships, and their relationship to salient features of their environment. HRQOL assessments were pioneered in the field of oncology [6] and, more recently, immunologists have transitioned and employed these tools to evaluate the impact and treatment of PIDD.

HRQOL assessments range from generic health status questionnaires that are applicable to all populations to more specific questionnaires that evaluate particular aspects of disease or treatment modalities. Generic HRQOL questionnaires are usually required for studies comparing impact of a specific illness to the general population. An example of a generic questionnaire is the Short Form-36 (SF-36). This self-administered tool contains 36 items and takes approximately 5 min to complete. It measures 8 dimensions of health in the areas of functional status, well-being, and overall evaluation of health [7, 8]. At the other end of the spectrum are disease specific measurements, which are used to evaluate more specific aspects of disease or treatment modalities. An example of this instrument is the life quality index (LQI). Each of these tools has been used to compare intravenous immunoglobulin (IVIG) and subcutaneous immunoglobulin (SCIG) therapies in PIDD patients [9, 10]. Some studies use a combination of these tools. Table 1 lists some of the most common QOL assessment tools used to evaluate PIDD and IgG replacement therapy.

**Table 1:** Common QOL assessment tools

HRQOL Indicator	Type of test	Health concepts measured	Advantages	Disadvantages
Child Health Questionnaire (CHQ-PF50) [12, 64, 65]	Designed to measure physical and psychosocial functioning and well-being. Aims to target both the child and the emotional impact of the child’s health on the parents Scores are converted to a 0–100 scale with higher scores indicating better functioning and well-being	Physical Functioning Role/Social-physical General health perceptions Bodily pain Parental time impact Parental emotional Impact Limitations in schoolwork or activities due to physical or emotional health Self-esteem Mental health General behavior Family activities Family cohesion Change in health	Strong feasibility and validity as well as consistency across clinical groups Widely used Available and valid in many languages	The application of CHQ in younger age groups requires adaptation Generic questionnaire, not able to provide a detailed measurement of dimensions that are specific for certain disease conditions
Short Form-36 (SF-36) [7, 8]	Generic health status form which contains 36 items evaluating an 8-scale profile of health concept measures of functional status, well-being and overall evaluation of health	Physical functioning Social functioning Physical problems Emotional problems Mental health Vitality Bodily pain General health perception	Brief, applicable to all populations and can be completed by individuals both with and without medical illness. Allows comparison across diverse groups, such as healthy and ill populations, or different age groups	Not disease specific Not a great amount of detail on each item
Pediatric Quality of Life Inventory (PedsQL 4.0) [13, 66, 67]	23 item scale encompassing parallel child self-report and parent proxy-report A 5-point response scale is utilized and items are translated to a 0–100 scale	Physical functioning (8) Emotional functioning (5) Social functioning (5) School functioning (5)	Maintains consistency across a broad age range of respondents and generally has a high level of feasibility and reliability	
Lansky’s Play Performance Scale (LPPS) [66, 68]	Observational scoring scale for children based on level of play and activity designed based on usual activities of the child, applicable across age groups and easily scored with ratings made by the parent	Based on a 10 point incremental scoring system which ranges from fully active (100) to unresponsive (0)	Good reliability and validity and widely used in pediatric oncology	Limited in that it looks only at functioning
Life Quality Index (LQI) [9, 10]	Developed to evaluate primary immunodeficiency patients on IVIG therapy and subsequently used in SCIG treatment Comprised of 15 items with assessments made on a 7-point Likert scale ranging from extremely good (= 7) to extremely bad (= 1)	Convenience Pain Improved health Interference with social/family life Interference with work/school Given in a comfortable place Does not require excessive wait time Given in a pleasant atmosphere Opinion on worth Anxiety/nervousness Expense Dependence on others Travel time and cost Limitations on my freedom Scheduling convenience	More sensitive to specific treatment-related changes in HRQL	Data cannot be compared outside of specific disease setting

The challenge of selecting and comparing QOL assessment tools is the variation in patient populations (children, adults, elderly, patients with comorbidities) and the applicability of individual tools to these specific patient populations. The Pediatric Quality of Life Inventory (PedsQL) and the Child Health Questionnaire (CHQ-PF50) are both widely used and valid generic measures in children, as is the SF-36 in adults [12, 13]. In a study assessing the PIDD-specific LQI in comparison to the generic CHQ-PF50 and SF-36, it was found that related scales correlated well though the LQI had internal consistency issues with measuring treatment costs [11]. There is still no universally accepted single best HRQOL measurement tool but continued efforts are being made to set quality criteria for these questionnaires [14, 15]. A strategy to combine generic and disease-specific measures in assessing PIDD HRQOL is ideal.

### QOL in all PIDD patients

In comparison with healthy children and adults, patients with PIDD experience measurably lower general health with higher hospitalization rates and increased physical, school, and social activity limitation [11, 16–20]. Even compared to patients with other chronic diseases, patients with PIDD score lower with respect to perception of general health [19, 20]. A six-year longitudinal study of patients with common variable immunodeficiency (CVID), a common form of PIDD, found that they scored lower on the General Health scale in comparison to other patients with chronic diseases, and that they had a worse perception of their general personal health and limitations due to their physical health [19]. This same study also found that PIDD patients’ QOL changed over time; in the 6 years between the first and final assessments, a significant decline was noted in scores relating to bodily pain, general health, limitation due to emotional problems, and limitation in physical and social functioning. Notably emphasizing the importance of patient HRQOL, this study found that patients’ relative risk of death, independent of age, was affected by their perceived physical and social function; each point increase in Physical and Social functioning scores reduced the risk of death by 2 and 3 % respectively [19]. Another study comparing children with PIDD with children with other chronic diseases found that they generally experience similar HRQOL to patients with juvenile inflammatory arthritis (JIA) but that they score lower than the JIA group with respect to perception of general health and limitations on parental time and family activities [20]. Mental health impacts have also been noted; children with PIDD found that they had significantly lower emotional and social functioning compared to children with JIA, as well as significantly higher rates of parent-rated depressive and anxiety symptoms [16].

A long delay in diagnosis and a large number of infectious episodes can further negatively impact HQROL [21] as well as lead to permanent functional impairments prior to initiation of treatment [22, 23]. A recent large scale analysis of a cohort of 2212 patients with CVID found that each year of diagnostic delay and each year of increased age at diagnosis were associated with a 1.7 and 4.5 % increased risk of death, respectively [24]. The median delay in diagnosis of PIDD has decreased considerably over the past 2 decades. A United States population-based cohort study found a 17 year median delay in diagnosis for patients whose symptoms occurred before 1986; this decreased to a 6.7 year median delay from 1986 to 1996, and decreased even further to a 2.7 year delay for patients whose symptoms presented after 1996 [25]. Despite these improvements, delayed diagnosis remains a significant problem [22, 26]. In a study of 32 patients with PIDD, more than two-thirds experienced diagnostic delay, which led to serious morbidity [27]. One study revealed that these patients experienced two major infections requiring hospitalization that included pneumonia, meningitis, osteomyelitis, and septicemia during this time of diagnostic delay [22].

Other factors have also been noted to influence HRQOL in patients with PIDD; a survey of 55 patients ≥20 years of age with PIDD found that quality of life scores were lowest in patients who were unemployed, had infections in more than four organs, had more than two additional diseases, and had experienced more than two specific occurrences of stress in the last 2–3 months [28]. Additionally, female gender, older age, chronic lung disease, and chronic diarrhea have also been shown to be risk factors leading to a poor quality of life in PIDD patients [19]. Some of the pertinent studies examining HRQOL in PIDD patients are summarized in Table 2.

**Table 2:** Examples of studies examining QOL in PIDD patients

Study	IVIG, SCIG or both	Adult or pediatric patients	Number of patients	Tools used	Summary
*Quality of life in PIDD Patients*					
Al-Herz et al. [69]	IVIG	Pediatric	98	LPPS	Patients with PIDD had poor performance status. Variables shown to have a positive effect on LPPS score included antimicrobial prophylaxis and IVIG therapy
Tcheurekdijian et al. [70]	IVIG	Adults	58	SF-36	Compared with age and sex matched diabetic and congestive heart failure patients, CVID patients on IVIG had significantly lower scores in general health and mental health. Compared with diabetic patients, CVID patients scored lower across all subscales with statistical significance in 6 of the 8 domains
Zebracki et al. [20]	IVIG	Pediatric	72	CHQ-PF50	Compared with age matched normal controls, PIDD patients on IVIG reported greater limitations in physical functioning, school related and social activities, general health, parental time emotional distress and limitations in family activities
Soresina et al. [18]	IVIG	Pediatric	25	PedsQL 4.0	X-linked agammaglobulinemia children had a significantly lower total score of HRQOL, psychosocial health, emotional functioning, social functioning and school functioning compared to healthy controls
Aghamohammadi et al. [21]	IVIG	Adults	36	SF-36	PIDD patients scored lower in physical and mental components compared to healthy controls. A reverse association was also noted between SF-36 scores and number of infectious episodes and long delays in diagnosis
Tabolli et al. [19]	Both	Adult and pediatric	96	SF-36; GHQ-12	CVID patients scored lower on the GHQ-12 than patients with other chronic diseases. Over 6 years of longitudinal assessment, a decline in the score on SF-36 scales was observed between the first and the last assessment for the Physical Functioning, Body Pain, General Health, Social Functioning, and Role-Emotional scales. The relative risk (RR) of death associated with PF and SF scales were noted to be 0.98 and 0.97 respectively, independent of patient age
*IVIG in Home versus Hospital/Doctor’s Office*					
Daly et al. [9]	IVIG- home based, self-infused	Adult and Pediatric	66 total 37 home 29 clinic	LQI (Daly); HOS; MHLC	Home based IVIG group revealed improved independence, convenience, comfort, decreased disruption of activities, travel time and costs
Espanol et al. [61]	IVIG, SCIG	Adult and Pediatric	300 total 160 IVIG 134 SCIG 6 other IG 150 home	SF-12; EQ-5D	Patients and caregivers prefer home treatment and less frequent self-administration of shorter duration with fewer needle sticks. Respondents receiving IVIG and those receiving SCIG had similar results though respondents receiving SCIG had a relatively higher preference for self-administration
*IVIG versus SCIG*					
Berger et al. [71]	SCIG previously on IVIG	Adult and pediatric	51	SF-36; CHQ-PF50	Adult patients had improvement in general health perception, vitality and mental health at 6 months and general health perception at 12 months. Children had improvements in general health at 6 and 12 months
Fasth et al. [72]	SCIG previously on IVIG	Pediatric	12	CHQ (Swedish version)	Patients and the parents of patients receiving SCIG had improvements in child-rated global health and social limitations subscale scores and parent-rated mental health, child’s general health and emotional impact and had significantly fewer days off school and workdays
Fasth et al. [73]	SCIG previously on IVIG	Pediatric	12	CHQ (Swedish version)	Parents noted significant improvements at 6 months in the child’s mental health, a change in health from 1 year ago and family activities. From the children’s perspective at 6 months there were significant improvements in global health and in role limitations and emotional social limitations
Hoffman et al. [39]	SCIG Previously on IVIG	Adult and pediatric	30	SF 36; CHQ-PF50	There was a significant improvement in general health perceptions, parental impact-emotional, parental impact-time, and family activities. At follow-up, 92 % of adults stated a preference for SCIG over IVIG therapy and 83 % preferred home therapy over therapy in clinic setting
Nicolay et al. [10]	SCIG vs IVIG	Adult and pediatric	58 (47 on IVIG prior to study)	LQI (Daly); CHQ-PF50; SF-36	In adults there was significantly increased satisfaction in scales of treatment interference and therapy setting in patients switching from IVIG to SCIG
Vultaggio et al. [52]	SCIG previously on IVIG and alternate SCIG	Adult and pediatric	40 (44 on IVIG prior to study, 6 on alternate SCIG prior to study)	LQI; CHQ-PF50; SF-36	In patients changing from IVIG to SCIG, no significant difference was seen in the SF-36 for patients over 14 years old or in the CHQ-PF50 answered by the caregivers of patients less than 14 years old but in both groups total mean LQI significantly improved at 6 months and the improvement was sustained over the 24 month follow-up period

*CHQ-PF50* Child heath questionnaire parental form 50, *EQ-5D* EuroQoL 5 Dimensions, *GHQ-12* General Health Questionnaire, *LQI* Life Quality Index, *LPPS* Lansky’s Play Performance Scale, *SF-12* Short Form 12, *SF-36* Short Form 36

* p < 0.05 for all values

### IVIG vs no therapy

For decades, replacement therapy with IgG has been the therapeutic cornerstone for antibody-deficient patients who have recurrent infections and are deficient in total or specific IgG. Despite the fact that IgG replacement therapy is the standard of care in many patients with PIDD, there are limited randomized controlled trials comparing IgG treatment with no treatment or placebo due to obvious ethical concerns; similarly, there is limited information on mortality [29, 30]. One study of patients with PIDD showed the proportion of persons surviving 10 years after diagnosis was 93.5 %, which is similar to the survival rate for the population at large [25]. It is known that IgG replacement therapy decreases the amount of life-threatening infections and studies have shown that there is a reduction in the rate of bacterial infections, days of antibiotic usage, days of fever, and hospital admissions following IgG therapy [31–35]. Abdou et al. found patients on IVIG therapy had a significant improvement in HRQOL and reduced number of infections after comparing a short 3-month period without treatment with 12 months of IVIG treatment [36]. In addition, it has been documented that maintaining adequate IgG levels can decrease infections and improve QOL [37–39].

### Routes of administration-SCIG vs IVIG

Many patients with PIDD require life-long IgG replacement therapy, thus the route of administration has many implications on patient well-being. The first case report of a patient treated with IgG replacement therapy was in 1952 and, interestingly, this patient was treated by SC infusion with favorable results [40]. The use of IgG replacement therapy has since become the standard of care for patients with PIDD. For many years, IVIG therapy in an outpatient setting was the treatment of choice. Then in the 1980s, slow SCIG infusions using portable syringe drivers were first introduced in the United States in the 1980s [41] followed by several other reports from the United States, Europe, and New Zealand [42]. IgG replacement therapy is now commonly begun by IVIG loading for three or more doses followed by a transition to SCIG. A large European database study included 7350 patients with PIDD and found that though 42 % of patients were on IgG replacement with IVIG being the most common form (75.8 %), followed by SCIG (23.9 %), and then intramuscular immunoglobulin (IMIG) (0.3 %). IMIG is the least widely used due to the pain associated with administration [43]. A 2008 survey of patients with PIDD conducted by the Immune Deficiency Foundation had similar findings with 69 % of patients in the sample using IVIG and another 23 % using SCIG [44].

Though IVIG appears to be the most common form of IgG replacement, SCIG treatment has shown promising results. One study using SCIG loading (100 mg/kg/day for 5 consecutive days) instead of IVIG loading at treatment initiation revealed that this was well-tolerated with no serious adverse events [45]. A recent review of three clinical efficacy and two extension studies found Hizentra^®^, a form of SCIG, to have comparable efficacy in comparison to IVIG in the prevention of serious bacterial infections and the maintenance of IgG levels [46]. Other reviews have also found that patients on SCIG clearly achieve acceptable IgG serum levels [37, 47] with minimal risk of systemic adverse events, including anaphylaxis [47].

A recent review of 25 studies noted that 4 studies evaluating health economics in SCIG and IVIG therapy all found SCIG to be more cost effective, mainly through the reduction of lost work or school days [48]. Other studies have shown that SCIG is more cost effective due to issues with IVIG such as personnel required to administer, facility rooms, administrative overheads and productivity loss [42, 49, 50]. In comparison to patients on IVIG, patients on SCIG miss significantly fewer days of work or school [51, 52] and one study also noted they spend significantly fewer days in the hospital [51]. PIDD patients’ perceived HRQOL are also affected by their choice of treatment [41, 52–54]. A Swedish study involving 25 PIDD patients, most of whom were on IVIG therapy, reported that initiating SCIG significantly increased their health-related function and improved self-rated health [54]. Similarly, a recent study of 50 PIDD patients shifting from IVIG to SCIG found that these patients subsequently had a significant improvement in their total mean LQI at 6 months that was sustained over time [52]. However, this same study acknowledges that it is unclear whether the LQI score improvement is due to the SCIG therapy itself, the accompanying switch to home treatments, or some combination of both factors. The importance of therapy convenience is also suggested by a past randomized crossover study comparing SCIG to IVIG administration; in this study, patients on SCIG were required to visit the hospital more frequently than those on IVIG therapy (once per week in comparison to once every 3–4 weeks) and only 10 of these 26 patients chose to continue with SCIG [55]. In fact, rather than SCIG therapy itself, a 3 year study in Norway noted that parents of children with PIDD actually cited greater independence and the ability to stay at home as the most beneficial results of switching from IVIG to SCIG [53]. The review of 25 studies also noted that, though transition from hospital-based IVIG to home-based SCIG therapy improved HRQOL, this improvement seemed to be largely related to home therapy [48].

### Home versus doctor’s office/hospital

Both SCIG and IVIG therapy are available for home administration, though the actual rate of use for each form of therapy differs. Of the PIDD patients surveyed in 2008 by the Immune Deficiency Foundation, 42 % of the patients on IVIG reported that they usually received their infusion at home and only 7 % were able to self-infuse; in comparison, nearly all SCIG users (93 %) received their treatment at home and were able to self-infuse [44]. Multiple studies have evaluated patient preferences regarding IgG replacement therapy at home versus in the doctor’s office or hospital. In patients treated with IVIG, Daly et al. found that home based IVIG improved independence, convenience and decreased disruption of activities [9]. Other studies have also shown that home-therapy regimens lead to greater improvement in HRQOL and treatment satisfaction [9, 11, 56, 57]. In patients treated with SCIG who had previously been treated with IVIG, Nicolay et al. investigated the impact of weekly SCIG self-infusions at home on quality of life and patient satisfaction [10, 56]. Patients who had previously been treated at the hospital/doctor’s office reported fewer limitations in their work/daily activities, significantly improved vitality, generally better health, and improved treatment satisfaction. The preference for the SC route and home therapy was 81 and 90 % respectively. In patients who were previously treated at home with IVIG, 69 % of patients preferred the SC route and 92 % preferred home therapy [56]. In an alternate study, 73 % of adult patients preferred SCIG over IVIG therapy, and correspondingly 82 % preferred home over hospital therapy; in children, 100 % preferred both SCIG and home therapy [11]. Notably, of the patients who had previously been treated with IVIG home therapy but later transitioned to SCIG home therapy, the majority preferred SCIG therapy after 1 year.

Patients’ ability to self-administer therapy at home has also been shown to impact their HRQOL. The 2008 Immune Deficiency Foundation Survey found that, while only 7 % of patients receiving home IVIG were able to self-infuse, nearly all patients receiving home SCIG were able to self-infuse [44]. Multiple studies have found that the ability to manage replacement therapy by oneself at home increases patient/family empowerment, makes it easier to work or study, improves family relations, contributes to a sense of self control among patients and families, and improves the daily living situation [53, 58–60]. Recently, a survey of 300 patients and caregivers of patients receiving IgG replacement (53 % receiving IVIG and 45 % receiving SCIG) found that patients and caregivers both preferred self-administration over administration by a health care professional [61]. This study again seems to suggest that convenience plays a large role in patient preferences, also noting that patients prefer home administration, less frequent administrations of shorter durations, and fewer needle sticks. In a German study, apprehension about self-infusion and a fear of side effects at home were cited as the main reasons that patients chose to remain on IV therapy [62]. Other factors that play into the decision of treatment location include accessibility to an infusion center, the patient’s schedule and availability during the hours that the infusion center is open, reliability of the patient as assessed by the physician, ability of the patient to learn and perform the techniques used in SCIG, safety, security, and cleanliness of the home environment, and issues related to reimbursement versus out-of-pocket costs [63]. Table 3 lists factors that positively and negatively impact HRQOL in PIDD.

**Table 3:** Positive and negative impact factors on HRQOL in patients with PIDD

Factors negatively impacting quality of life in PIDD patients
Delay in diagnosis
Other chronic health issues
Stress
Unemployment
Repeat infectious episodes
Social factors such as unemployment
Chronic lung disease

Factors positively impacting quality of life in PIDD Patients
Treatment in the home setting
Independence
Convenience of treatment
Comfort of treatment
Less parental impact time
Therapeutic IgG trough levels

## Conclusion

PIDD with significant hypogammaglobulinemia is typically characterized by recurrent and severe bacterial infections of the upper and lower respiratory tracts and is associated with poorer HRQOL than patients without chronic illness. Increased knowledge of specific immunodeficiencies has led to a rise in the diagnosis and incidence of PIDD. Since patients with PIDD often require life-long treatment with IgG, choices about the route of administration have a significant impact on HRQOL. Choosing the right patient, providing proper support, and managing expectations are key to ensuring that patients with PIDD achieve the maximum benefit from therapy.

## Abbreviations

CHQ-PF50: child heath questionnaire parental form 50
HRQOL: health related quality of life
IMIG: intramuscular immunoglobulin
IVIG: intravenous immunoglobulin
JIA: juvenile inflammatory arthritis
LPPS: Lansky’s play performance scale
LQI: life quality index
PIDD: primary immunodeficiency disease
SCIG: subcutaneous immunoglobulin
SF-36: short form 36
